# Near Real Time EHR Data Utilization in a Clinical Study

**DOI:** 10.3233/SHTI200178

**Published:** 2020-06-16

**Authors:** Melody L. PENNING, Collette BLACH, Anita WALDEN, Pei WANG, Katrina M. DONOVAN, Maryam Y. GARZA, Zhan WANG, Julie FRUND, Shorabuddin SYED, Mahanazuddin SYED, Guilherme DEL FIOL, L. Kristin NEWBY, Carl PIEPER, Meredith ZOZUS

**Affiliations:** a University of Arkansas for Medical Sciences, Little Rock, AR, USA; b Duke University Molecular Physiology Institute, Durham NC, USA; c Depart of Biomedical Informatics, University of Utah, Salt Lake City, UT, USA; d Duke Clinical & Translational Science Institute, Duke University Medical Center, Durham, NC, USA; e Department of Biostatistics & Bioinformatics, Duke University, Durham, NC, USA; f University of Texas Health Science Center San Antonio, San Antonio, TX, USA

**Keywords:** Data integration, clinical trials, data quality, data processing

## Abstract

Extraction and use of Electronic Health Record (EHR) data is common in retrospective observational studies. However, electronic extraction and use of EHR data is rare during longitudinal prospective studies. One of the reasons is the amount of processing needed to assess data quality and assure consistency in meaning and format across multiple investigational sites. We report a case study of and lessons learned from acquisition and processing of EHR data in an ongoing basis during a clinical study.

## 1. Introduction

Large sets of electronic health record (EHR) data present an exciting possibility for research such as epidemiologic and observational research, real-time data collection, safety surveillance and regulatory uses and prospective clinical research. [3,4,6] Although this data has much potential, there are many issues that must be addressed in order to produce consistent and reliable findings. [10] EHR data are data collected in the process of care. Since the primary purpose of EHRs is to support activities such as clinical workflow and billing, the resulting data format, structure and quality often differ from the carefully scheduled, collected and formatted data required for research. In addition, EHR data changes over time reflecting (1) changes in the health status of the patient, and (2) changes in technology, data representation formats, regulatory incentives and institutional factors. [5] These variations in expected data versus collected data (e.g., timeliness, accuracy) are collectively referred to as data quality dimensions. [8,11]

EHR data provide useful information about a patient and their healthcare only if the data quality is high and correspond to the real world. This correspondence varies widely, as do the measurement methods employed in the published literature. [1; 9] Further, the reasons for observed inconsistencies are rarely reported. To address this deficit in our understanding of data quality in healthcare data, the Patient Centered Outcomes Research Institute (PCORI) EHR Data Quality Study compares EHR and patient self-reported data for 34 conditions, 8 medical procedures, hospitalizations, and smoking status in cohorts from two southern U.S. states (Arkansas and North Carolina). This report outlines the data acquisition and processing of EHR data for this study. Briefly, the study required near real time acquisition and processing of EHR data to facilitate the comparison of EHR with self-report data to (1) identify discrepancies between these sources, and (2) support subsequent EHR data investigations and patient interviews on a weekly basis.

## 2. Methods

We report on the common aspects of data acquisition and processing between the two regions. Inpatient data from large hospitals were extracted from institutional data warehouses. Data from outpatient clinics were obtained either directly from the clinic EHR (North Carolina) or from an institutional data warehouse (Arkansas). Patient self-report data were compared with phenotypes derived from EHR data for the conditions, procedures, hospitalizations and smoking status from Arkansas and conditions from North Carolina.

For each phenotype, computational algorithms were developed to render a “self-report” answer and an “EHR answer”. Each was classified as “yes/confirmatory”, “don’t know/possible”, or “no/no evidence” in the self-report and EHR data respectively. Mismatches between EHR and self-reported phenotypes were considered a discrepancy. A subset of study participants were interviewed about their discrepancies. In the Arkansas cohort, participants were prospectively enrolled and the study required acquisition and processing of data on a weekly basis. In North Carolina, participants from a longitudinal community registry and biorepository, the Measurement to Understand Reclassification of Disease of Cabarrus/Kannapolis (MURDOCK) Study, have provided self-report data with annual updates as well as consent to access their EHR data. [2]

## 3. Results

For the North Carolina cohort, self-report data were managed in a custom-developed system. Existing self-report data were compared to newly acquired EHR data and discrepancy reports. For the Arkansas cohort, participants were prospectively enrolled at six clinics throughout the state. In Arkansas, self-report data were collected only once for the purpose of the PCORI EHR Data Quality Study. Data for the Arkansas cohort were managed in the open-source Community Edition of the OpenClinica Electronic Data Capture (OC-EDC) system. Discrepancies for both cohorts were double coded for root cause and correct data source in the OC-EDC system. The orchestration of these sources was accomplished with carefully timed data transfers and SQL queries running over the entirety of the combined patient populations with 1,061,999 patients and 23,839,223 encounters.

Research Staff had real time access to EHR Data. For the Arkansas cohort, participant healthcare data were captured in either the Epic^®^ EHR or various versions of Centricity^®^. These data were directly accessed through the EHR user interface by the research assistants (RAs) at enrollment to obtain participant medical record numbers (MRN) to for patient matching between self-report and EHR data. The RAs also used Epic to look up diagnostic information (1) prior to the telephone discrepancy interview when a discrepancy appeared likely due to clinical charting under the wrong patient or record linkage errors; and (2) for post interview attempts to confirm root cause. Because the study was operationalized differently in North Carolina due to previously existing EHR and self-report data, and because the clinics were separate institutions from the MURDOCK Study, EHRs in North Carolina were not accessed through the EHR user interface.

Acquisition of EHR data required substantial preprocessing. For the Arkansas cohort, all data were extracted from the institutional data warehouse for enrolled participants. The extracted data were stored in Microsoft SQL server and required several layers of conversion processing in order to integrate the regional program data with the hospital data. For the North Carolina cohort, EHR data were obtained from five local healthcare facilities with the following EHRs: Athena Health^®^, Allscripts^®^, Practice Fusion^®^ and Epic^®^. The data from the different facilities were standardized and transformed to a common data model by the MURDOCK Study team. These data were linked with the self-report data, de-identified and sent to the study coordinating center for further processing. Record linkage between the incoming self-report data and EHR data was a deterministic match for both cohorts using the MURDOCK Study Number (North Carolina) and the MRN (Arkansas). Any linkage errors discovered in the EHR were collected as part of the study.

Following record linkage, each participant’s EHR data were phenotyped. Phenotypes are rule-based definitions used for classification. Combinations of standardized controlled coded terminologies for healthcare (the International Classification of Diseases (ICD) and Current Procedural Terminology (CPT)), along with a few other non-coded data points identified from the existing literature and from practicing physicians, were used to develop the rule- based definitions. These were run over the entire dataset.

The phenotyping process was completed using stored procedures and each processing step was logged in case of failure. Overall, 140 phenotypes (70 “confirmatory” and 70 “possible”) were specified and programmed. A total of 7,405 unique ICD-9 and ICD-10 codes were used in the process. Due to the long list of codes, a flexible design was necessary. Value sets for all phenotype definitions were kept in one table to be read by stored procedures, providing a quick and easy way to address unexpected phenotype results with simple table insertions/deletions as opposed to time consuming and risky code alterations. Records returned from the EHR for each phenotype run were compared with those from the self-report dataset. Each condition, procedure and event of interest for each participant was classified as “discrepant”, “possibly discrepant” or “not discrepant”. Discrepancies were then output to reports for RAs to use in the interview process. In addition to the discrepancy details, supplemental information such as ICD code descriptions were included to help characterize each discrepancy. Discrepancies were also imported into the OpenClinica system for post- interview coding (root cause and most likely correct data source) and adjudication. Finally, coded and adjudicated discrepancies were exported from the OpenClinica system back to the Microsoft SQL Server for final post-interview discrepancy reports which were made available to participants.

To date, 2,151 participants have been enrolled in Arkansas. Of those, 1,096 participants have been successfully processed through discrepancy identification. Of the North Carolina cohort, 3,927 MURDOCK Study participants had EHR data and have been processed through discrepancy identification, of which 373 participants have been interviewed. The study is ongoing at this time.

## 4. Discussion

The lack of information about the level of correspondence between healthcare data and the real world is an impediment to various types of research. Data quality must not be an unknown. The PCORI EHR Data Quality Study is an effort to test dimensions of healthcare data quality over multiple phenotypes simultaneously in order to reveal data quality issues at scale and establish general measures and methods for characterization of data quality problems in health record data.

Modifications were needed in response to both internal and external changes. Some phenotypes were especially challenging, such as obesity, kidney disease and chronic obstructive pulmonary disease (COPD). Initially phenotypes were developed with broad definitions and code sets. Through iterations of ICD code tuning they were refined to more conservative sets that reflected the intent of the phenotype. In addition, an inherent dependency on data source systems provided challenges. Changes to institutional software along with a national migration from ICD version 9 to version 10 affected our design and led to reanalysis, redefinition and reprogramming. During system development, a new EHR system for the Arkansas regional clinics was implemented which required additional programming to access the data that had not yet been integrated into the institutional data warehouse. Additionally, some specific ICD codes were also updated during this time period. System changes are inevitable, as institutions maintain EHR systems and controlled terminologies evolve. Due to flexibility of the system design, we were able to resolve phenotype tuning issues and adapt to external and internal environmental changes smoothly.

Several concrete lessons were learned that are unique to comparing EHR with self-report data. First, record linkage can be problematic, even in cases where id numbers are used. [7; 8] Linked data should always be fact checked to make sure errors have not occurred. Second, phenotype definitions will evolve and change based on data source, date of collection and workflow or process of collection. Third, comparisons for events will cause a multiplicity of “no match” conditions (e.g. hospitalizations and procedures can easily lead to a large *all possible combinations* problem if the date windows are not sufficiently tolerant). Lastly, the permutations of possible outcomes (discrepant, possible, and not discrepant) must be very carefully considered. These conditions were discussed on multiple occasions as new information came in from phenotype runs and were compared directly against data in Epic^®^.

This work has provided a window into the quality of EHR data through the design and implementation of a near-real-time data quality measurement system while supporting study operations for informatics and research. While the architecture was designed for the University of Arkansas’ environment it may be adapted and generalized to work at other institutions and for other data sources.

## 5. Conclusion

It is well-known that the quality of EHR data varies from institution to institution. The implementation reported here identified additional challenges in acquisition and assessment of discrepancies between EHR data and patient self-report data. Even with well-defined approaches a significant amount of effort to adapt and keep up with institutional changes and handle unpredictable quality issues was needed. The work reported here required a team- based approach with the technical staff, informaticists, clinicians and other subject matter experts. Observations and lessons learned from this implementation may inform others as they embark upon use of EHR data during and to support clinical and informatics studies.
